# Diatoms Reduce Decomposition of and Fungal Abundance on Less Recalcitrant Leaf Litter via Negative Priming

**DOI:** 10.1007/s00248-023-02268-w

**Published:** 2023-07-28

**Authors:** Alexander Feckler, Patrick Baudy-Groh, Lisa Friedrichs, Sara Gonçalves, Simon Lüderwald, Ute Risse-Buhl, Mirco Bundschuh

**Affiliations:** 1 iES Landau, Institute for Environmental Sciences, RPTU Kaiserslautern-Landau, Fortstraße 7, 76829 Landau, Germany; 2 Department of Aquatic Sciences and Assessment, Swedish University of Agricultural Sciences, Box 7050, 75007 Uppsala, Sweden; 3 Eußerthal Ecosystem Research Station, RPTU Kaiserslautern-Landau, Birkenthalstraße 13, 76857 Eußerthal, Germany; 4 Department of River Ecology, Helmholtz Centre for Environmental Research – UFZ, Brückstraße 3a, 39114 Magdeburg, Germany

**Keywords:** Stream ecosystem, Leaf litter decomposition, Priming effect, Periphytic algae, Heterotrophic decomposers, Labile carbon

## Abstract

Heterotrophic microbial decomposers colonize submerged leaf litter in close spatial proximity to periphytic algae that exude labile organic carbon during photosynthesis. These exudates are conjectured to affect microbial decomposers’ abundance, resulting in a stimulated (positive priming) or reduced (negative priming) leaf litter decomposition. Yet, the occurrence, direction, and intensity of priming associated with leaf material of differing recalcitrance remains poorly tested. To assess priming, we submerged leaf litter of differing recalcitrance (*Alnus glutinosa *[alder; less recalcitrant] and *Fagus sylvatica *[beech; more recalcitrant]) in microcosms and quantified bacterial, fungal, and diatom abundance as well as leaf litter decomposition over 30 days in absence and presence of light. Diatoms did not affect beech decomposition but reduced alder decomposition by 20% and alder-associated fungal abundance by 40% in the treatments including all microbial groups and light, thus showing negative priming. These results suggest that alder-associated heterotrophs acquired energy from diatom exudates rather than from leaf litter. Moreover, it is suggested that these heterotrophs have channeled energy to alternative (reproductive) pathways that may modify energy and nutrient availability for the remaining food web and result in carbon pools protected from decomposition in light-exposed stream sections.

**Supplementary Information:**

The online version contains supplementary material available at 10.1007/s00248-023-02268-w.

## Introduction

Up to 90% of the global terrestrial plant production enters the dead organic matter pool [1] and the decomposition of such organic matter represents one of the most important processes for the energy supply in terrestrial and aquatic ecosystems [2]. This process is accomplished among others by heterotrophic microbial decomposers such as fungi and bacteria [2]. These microorganisms colonize organic matter and convert low- and high-molecular-weight compounds into bioavailable mono- and disaccharides [3]. Among heterotrophic decomposers in aquatic ecosystems, especially the polyphyletic fungal group of aquatic hyphomycetes (AH) have evolved to decompose recalcitrant organic matter and do supply carbon (C) to the remaining food web. In contrast to AH, bacteria seem to play a less prominent role in this process [4]. When decomposing recalcitrant organic matter, heterotrophs are in close spatial proximity of autotrophs, potentially enabling the exchange of metabolic products [5]. For instance, periphytic algae produce exudates, rich in labile compounds such as carbohydrates and amino acids [6]. These algal exudates have been shown to affect heterotrophs’ growth and activity in positive and negative ways, also known as “priming” [7]. Despite being important for the global C cycle and being well understood in soil ecosystems, only few studies have assessed priming in aquatic ecosystems [e.g., 8–11]. Consequently, its importance for C turnover in lakes and streams remains poorly understood. More importantly, the outcome of studies on priming in aquatic ecosystems yielded contradictory results. Some studies [e.g., 8] observed positive priming, which refers to the heterotrophic use of labile organic C resulting in a higher decomposition [12]. Other studies [e.g., 11] showed negative priming, where leaf litter decomposition is reduced as microbial heterotrophs likely invest labile organic C rather to respiration, reproduction, or growth instead of enzyme production [9]. In addition, studies also reported no impact on heterotrophs due to priming [e.g., 9, 10].

The occurrence, direction, and intensity of priming in aquatic ecosystems seem to depend on various external variables. On the one hand, algae-mediated stimulation of C processing by heterotrophs may depend on nutrient concentrations in the surrounding media [13]. Low nutrient concentrations maximize the competition for inorganic nutrients among microbes, leading to reduced algal growth as they are outcompeted by heterotrophs [14]. At the same time, nutrient-limited algae increase their exudation of labile organic C [15], potentially stimulating priming effects. On the other hand, the recalcitrance of organic matter should mediate priming, leading to stronger effects associated with more recalcitrant than with less recalcitrant organic matter [11]. This is because heterotrophs should be more limited in their ability to obtain leaf-bound C when colonizing more recalcitrant organic matter, an assumption supported by studies in soil ecosystems [16].

Therefore, we investigated the effect of light and the individual and joint contributions of periphytic algae (i.e., diatoms), bacteria, and fungi (represented by AH) to microbially-driven leaf litter decomposition under low nutrient availability from the surrounding medium. We took contrasting C recalcitrance and nutrient content of the organic material into account by using two leaf species (black alder (*Alnus glutinosa* (L.) Gaertn.) and European beech (*Fagus sylvatica* (L.)) that strongly differ in their lignin and nutrient content. Under the assumptions described above, we hypothesized that the low availability of nutrients from the surrounding medium should result in (i) positive priming in microbial treatments containing all microbial groups in the presence of light, which leads to positive effects on the growth and activity of heterotrophs and by this increase leaf litter decomposition [13] and (ii) higher priming intensity associated with leaf litter of European beech than for black alder, given the higher recalcitrance and lower nutrient content of the former resulting in a stronger dependence on external C sources.

## Material and Methods

### Sources of Leaf Material and Microorganisms

Senescent but undecomposed leaves of black alder and European beech were picked from trees near Landau, Germany (e.g., N 49° 12′, E 8° 13′), during autumn 2018, and stored at − 20 °C. We deliberately selected the N-fixing and less recalcitrant species black alder as well as the N-poor and more recalcitrant species European beech as model leaf species because of the marked differences in their litter traits (alder: 12 ± 0.5 mg lignin g dw^−1^, 189 ± 3 mg nitrogen g dw^−1^, 7.0 ± 0.4 mg phosphorous g dw^−1^; beech: 26 ± 1 mg lignin g dw^−1^, 64 ± 2 mg nitrogen g dw^−1^, 2.1 ± 0.3 mg phosphorous g dw^−1^; [17]) and therefore conjectured differences in the priming intensities.

We used a *Nitzschia palea* strain (isolate TCC139-1) obtained from the Institut national de la recherche agronomique (INRA; Thonon-les-Bains, France), which was maintained for 4 weeks prior to the test commencement to allow acclimatization to the test medium (Table S1) and laboratory conditions. The nutrient content of the test medium was adjusted to 0.2 mg NO_3_-N L^−1^ and 0.02 mg PO_4_-P L^−1^, mimicking low environmentally relevant availabilities of essential nutrients for microbial decomposers [18, 19] that match well with those nutrient concentrations applied in earlier studies on priming [9, 13]. Laboratory conditions comprised a temperature of 16.0 ± 0.3 °C (mean ± standard error; *n* = 5; measured every 30 min over 30 days using data loggers; HOBO, MA, USA) and a 16:8-h light:dark rhythm, while the intensity of the photosynthetically active radiation (PAR) corresponded to the irradiance on streambeds during summer months (~ 40 µmol m^−2^ s^−1^ PAR) [20]. We exchanged the medium weekly during the acclimatization phase to ensure a constant nutrient supply. Cell densities in the cultures that served as a diatom stock at the test commencement were quantified fluorometrically [21]. Therefore, we established a calibration curve between cell numbers and fluorescence on a microplate reader (Tecan Infinite® M200; Tecan Group, Mänedorf, Switzerland; excitation, 430 nm; emission, 680 nm).

We obtained bacteria from the near-natural stream Hainbach (Germany; N 49° 14′, E 8° 03′), by collecting 1 L of stream water and in-stream leaf material in a sterilized glass bottle one day before test commencement. In the laboratory, we used a sterilized filtration system to pass stream water through sterile glass fiber filters (GF/C, pore size 1.2 µm; Whatman, NJ, USA). Other unicellular organisms with a size smaller than 1.2 µm (e.g., archaea) only contribute to a minor share to the microbial biomass in the solution, justifying our assumption to mainly work with a diverse bacterial community in solution [22, 23]. An aliquot of this bacterial solution was further sterilized by filtration through Isopore™ membrane filters (pore size 0.2 µm; Merck Millipore, Darmstadt, Germany) and steam autoclaving at 121 °C for 15 min for the use in treatments without bacterial presence (Table 1). Both the bacterial and the sterilized solutions were kept in sterilized glass bottles at 4 °C under permanent stirring until their use in the experiments (max. 12 h).

**Table 1:** Overview of the applied microbial treatments, which were tested in absence and in presence of light (photosynthetically active radiation)

Microbial treatment	Initial microbial community in each replicate
Diatoms low	~ 1000 *Nitzschia palea* cells, 1 mL of sterilized bacterial solution, and 6 sterile agar plugs
Diatoms high	~ 10,000 *N. palea* cells, 1 mL of sterilized bacterial solution, and 6 sterile agar plugs
Bacteria	1 mL of non-sterilized bacterial solution and 6 sterile agar plugs
Fungi	1 agar plug of each of the six aquatic hyphomycete (AH) cultures (up to 7.5 ng fungal biomass per species) and 1 mL of sterilized bacterial solution
Combined low	~ 1000 *N. palea* cells, 1 mL non-sterilized bacterial solution, and 1 agar plug of each of the AH cultures
Combined high	~ 10,000 *N. palea* cells, 1 mL non-sterilized bacterial solution, and 1 agar plug of each of the AH cultures

To generate an assemblage of six AH, we used in-house cultures of the species *Alatospora acuminata* Ingold, *Clavariopsis aquatica* de Wild., *Heliscella stellata* (Ingold & V.J. Cox) Marvanová, *Neonectria lugdunensis* (Sacc. & Therry) L. Lombard & Crous (formerly *Heliscus lugdunensis*), *Tetracladium marchalianum* de Wild., and *Tricladium angulatum* Ingold. The strains were isolated from German streams and are deposited at the Leibniz Institute DSMZ (German Collection for Microorganisms and Cell Cultures, Germany). AH cultures were maintained in axenic conditions on 2% malt extract agar in Petri dishes. Two weeks prior to the test initiation, we inoculated sterile 1% malt extract agars using 0.5 × 0.5 cm agar plugs from the growing front of the maintenance cultures for their use in the bioassays. We preserved samples of the AH cultures during test commencement to quantify the initial biomasses of the individual AH species used as inocula for the leaf material (see chapter “Analyses of leaf-associated microbial assemblages”; Table 1).

### Bioassays

We conducted two 30-days lasting bioassays, one for each of the two tested leaf species, using the same experimental approach. For each replicate (*n* = 15), we prepared a set of two leaf strips (~ 6 × 10 cm each) from thawed leaves. Leaf strips were leached in aerated ultrapure water for 48 h before further processing to avoid that microbially-driven leaf mass loss was confounded by the loss of soluble leaf components [24]. Afterwards, we sewed leaf strips of known dry weight (nearest 0.01 mg) into fine-mesh nylon gauze bands (~ 6.5 × 10.5 cm; 500 µm aperture) after brief re-soaking in ultrapure water to prevent leaf fragmentation.

Each set of leaf strips was introduced into an individual microcosm consisting of a sterilized 250-mL glass beaker filled with 225 mL of sterile test medium. Six microbial treatments were tested to investigate the effect of light and the individual and joint contributions of diatoms, bacteria, and fungi (represented by AH) to microbially-driven leaf litter decomposition (Table 1). Depending on the microbial treatment, each microcosm received ~ 1000 or ~ 10,000 *N. palea* cells, 1 mL of the bacterial solution, and/or 1 agar plug (Ø 5 mm) from each of the six AH cultures cut from the growing front. Treatments without bacterial and/or fungal presence received 1 mL of the sterilized solution and/or six sterile agar plugs (Ø 5 mm) per microcosm, respectively, to account for nutrient imbalances among treatments. Afterwards, we randomly set up the microcosms in an environmental test chamber set at 16.0 ± 0.3 °C, either in absence or in presence of light at a 16:8-h light:dark rhythm. Each microcosm was thoroughly aerated to create water turbulence and induce fungal sporulation [25].

Every tenth day, we transferred the leaf strips into 195 mL of fresh, sterile test medium amended with 30 mL of the old test medium from the respective microcosm to transfer labile organic C. Furthermore, each “diatom present” treatment (Table 1) received ~ 1000 or ~ 10,000 fresh *N. palea* cells, given that diatoms tend to stick to silica glass walls and were not necessarily transferred into the new microcosms [26]. Coinciding with each medium renewal, we destructively sampled five random replicates per treatment to determine leaf mass loss and microbial responses. The remaining dry weight (nearest 0.01 mg) of one leaf strip was quantified after drying for 24 h at 60 °C to calculate the microbially-mediated leaf mass loss [27]. The second leaf strip was used to cut leaf discs (Ø 16 mm) for analyses of the leaf-associated microbial assemblages.

### Analyses of Leaf-Associated Microbial Assemblages

Quantitative real-time polymerase chain reaction (qPCR) analyses were performed to estimate the leaf-associated DNA amounts of individual AH species and to estimate the numbers of leaf-associated bacterial and fungal operon copies used as proxies for bacterial and fungal abundance, respectively. Therefore, we extracted DNA from two leaf discs (Ø 16 mm) of known dry weight per replicate using the FastDNA^®^ Spin Kit for Soil and the FastPrep™-24 5G instrument (both MP Biomedicals, Schwerte, Germany). We included extraction controls and environmental controls in each extraction run to account for potential contamination of the samples. These controls showed no sign of the target DNA, indicating reliability of the results.

We estimated the leaf-associated DNA amounts of individual AH species as per Baudy et al. [28]. Undiluted extracts were used to quantify DNA of the model AH in species-specific TaqMan^®^ qPCR reactions (Applied Biosystems, USA). For a detailed description of the species-specific TaqMan^®^ assays, see Table S2.

We quantified leaf-associated bacterial and fungal operon copy numbers as proxies for microbial abundances [29]. The primer pairs E8F/E533R [30] and ITS3F/ITS4R [31] were used for bacterial and fungal analyses, respectively. We diluted the DNA extracts 50-fold and used the dilutions in group-specific SYBR™ Green qPCR reactions (Thermo Fisher Scientific GmbH, Dreieich, Germany). Calibration curves covering a gradient from 10^4^ to 10^9^ copies of “model amplicons” of the bacterium *Escherichia coli* (Migula 1895) Castellani and Chalmers 1919 and the AH *T. marchalianum* (both Thermo Fisher Scientific, CA, USA; Table S3) were run in parallel, which can be seen as external positive controls. We carried out melting curve analyses at the end of each qPCR run to test the specificity of the assays by initial denaturation for 15 s at 95 °C, followed by a steady temperature increase for 20 min from 60 to 95 °C (see Fig. S1 for an exemplary melting curve). For a detailed description of the respective assays, reaction compositions, cycling conditions, and data analysis, see Manerkar, Seena, and Bärlocher [29]. We performed all qPCR reactions on a Mastercycler^®^ ep gradient S (Eppendorf, Hamburg, Germany) using 0.2-mL 8-tube strips covered with clear optical 8-cap strips (Sarstedt AG & Co. KG, Nümbrecht, Germany). All results were dry-weight normalized to the respective leaf discs (nearest 0.01 mg).

In addition, diatoms associated with leaf material and diatoms present in the medium as well as associated with the glass walls of the microcosms were quantified by high-performance liquid chromatography (HPLC) using fucoxanthin as a biomarker [32]. We chose fucoxanthin as a biomarker for diatoms instead of the commonly used chlorophyll *a*, as the latter may have also originated from the leaf material leading to confounded results. In brief, leaf material was lyophilized for 48 h, homogenized (Beat Ruptor Elite; Omni International, GA, USA), and weighed to the nearest 0.1 mg. To sample diatoms present in the medium and attached to the glass walls of the microcosms, we scraped off the microcosms’ walls with a rubber policeman during medium renewal. Subsequently, we filtered the medium together with any scraped off material over pre-combusted (450 °C for 5 h) glass fiber filters (pore size 1–3 µm; GF/6; Whatman™, Germany). We extracted fucoxanthin from leaf material and the filters with 99% ethanol (two freeze–thaw cycles) as well as sonification. A filtered (pore size 0.45 µm; MACHERY-NAGEL™ CHROMAFIL™ PA; UK) subsample of 20 µL was analyzed by HPLC (Ultimate3000, Thermo Fisher Scientific Corporation, Waltham, MA, USA) [33]. Fucoxanthin concentrations were calibrated using standards from the DHI Water and Environment Institute (Hørsholm, Denmark).

### Data Treatment and Statistical Analyses

We calculated time-integrated bacterial and fungal abundances following the procedure of Soares et al. [10] by integrating the area under a fitted curve for each 10-day interval (i.e., the individual sampling points; R package “bayestestR” [34]) and multiplied the time-point rates by the respective time periods to calculate abundances. This procedure led to three estimates per microbial group, which were summed up to obtain the overall leaf-associated microbial abundances over the entire experimental duration. For the leaf mass loss, however, we only analyzed the data obtained after 30 days, as these data integrate the leaf mass loss of previous sampling points. We averaged the estimates for leaf-associated DNA amounts of individual AH species for each treatment and time point separately for each of the two assessed leaf species to reduce the quantity of data and facilitate their analyses (for data at the individual sampling dates, see Supporting Information).

We checked the normality of residuals and heteroscedasticity of the univariate data (all variables except AH assemblage composition) using quantile–quantile plots and Levene’s tests, respectively. Depending on the data, we applied generalized linear models (GLMs) with an assumed Gaussian or Gamma distribution of the response variable with an identity or inverse link function, respectively, to determine the statistical significance of the assessed factors (“leaf species”, “microbial treatment”, or “light condition”) and their interactions. To check the model fits and verify the validity of the assumed data distributions as well as link functions, we used the model diagnostics implemented in the R package “DHARMa” [35]. The effects of the factors as well as their interaction on the dependent variables were analyzed with type III ANOVAs. Statistically significant differences among microbial treatments under the two light conditions were assessed individually for the two leaf species using Tukey’s test for post hoc analysis using the R package “emmeans” [36].

However, given the ongoing debate on the application and interpretation of null hypothesis significance testing [37], we additionally calculated Bayes factors (BF). BF offer information that allows statements about the likelihood of the alternative hypothesis, rather than just the null hypothesis, and provide a clearer estimate of the amount of evidence present in the data [38]. Compared to a null hypothesis significance testing approach, the Bayesian approach to hypothesis testing is comparative in nature, meaning that the likelihood of the data under both the null model and the alternative hypotheses is considered, and those probabilities are compared through the BF. In other words, the BF_10_ can be interpreted as the ratio that contrasts the likelihood of the data under the alternative hypothesis (*H*_*1*_) with the likelihood of the data under the null hypothesis (*H*_*0*_). Consequently, there is more evidence in support of the alternative hypothesis over the null hypothesis as BF_10_ increases [39]. We calculated BF_10_ for the assessed factors and their interactions using the R package “BayesFactor” [40]. The interpretation of BF as evidence for the alternative hypotheses compared to the null hypotheses (BF_10_) followed the terminology of Jarosz and Wiley [39].

For multivariate data (AH assemblage compositions), we checked the multivariate homogeneity of group dispersions (i.e., variances) using the “betadisper” function in the R package “vegan” [41]. Next, we determined the statistical significance of the assessed factors and their interactions with a permutational multivariate analysis of variances (PERMANOVA), performed on square-root-transformed data to reduce the effect of dominant groups [42] and applying Bray–Curtis dissimilarities as distance measure between the groups. In addition, we prepared nonmetric multidimensional scaling (NMDS) ordination plots [43] individually for the two tested leaf species for a graphical representation of the observed dissimilarities. Finally, we applied similarity percentages analyses (SIMPER; implemented in the R package “vegan”) to identify AH species that primarily explain the observed differences between leaf species.

We used the open-source statistical software R [44] supplemented by the required add-on packages for data analyses and preparation of figures. The level for statistical significance was set at *p* < 0.05 and the term “significant(ly)” is exclusively used in the sense of “statistical significance”.

## Results and Discussion

**Fig. 1:**
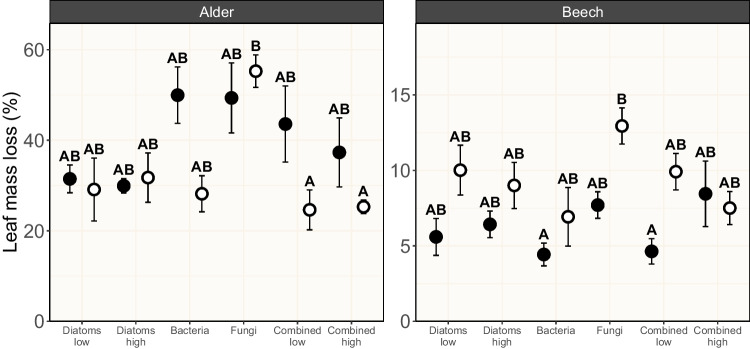
Mass loss (%; mean ± standard error [SE]; *n* = 5) of black alder (left) and European beech (right) leaf material after 30 d of being subjected to six microbial treatments in absence (filled circled) and in presence (open circles) of light. Letters above error bars denote results of pairwise comparisons among treatments, with different letters indicating statistically significant differences among treatments. Details on the microbial treatments are given in Table 1

Microbially driven leaf mass loss was very strongly and decisively impacted by the light condition (*χ*^2^ = 5.90, *p* = 0.015, BF_10_ = 93.98) and the leaf species (*χ2* = 87.99, *p* < 0.001, BF_10_ > 150), respectively, while these two factors interacted (*χ*^2^ = 9.38, *p* = 0.002, BF_10_ > 150; Table 2). The presence of light generally reduced alder decomposition while increasing beech mass loss in most of the assessed microbial treatments (Fig. 1). At the same time, leaf mass loss was substantially higher for alder leaves across all microbial treatments when compared to beech leaves, which is in accordance with earlier studies [45]. The microbial treatment, on the other hand, showed only an anecdotal effect on the leaf mass loss of the two leaf species, irrespective whether assessed individually or in combination with the other factors (*χ*^2^ ≥ 8.47, *p* ≥ 0.023, BF_10_ ≤ 1.76; Table 2).

**Table 2:** Output for the generalized linear models and Bayesian ANOVAs testing the effects of leaf species (S), microbial treatment (M), and light condition (L) on leaf mass loss, bacterial abundance, and fungal abundance. The interpretation of Bayes factors as evidence for the alternative hypotheses compared to the null hypotheses (BF_10_) followed the terminology of Jarosz and Wiley [39]

Response	Factor	*χ*^2^-value	*p*-value	BF_10_	Evidence for alternative hypotheses
Leaf mass loss	S	87.99	< 0.001	> 150	Decisive
	M	8.84	0.116	1.76	Anecdotal
	L	5.90	0.015	93.98	Very strong
	S × M	13.03	0.023	0.52	Anecdotal
	S × L	9.38	0.002	> 150	Decisive
	M × L	8.76	0.119	0.61	Anecdotal
	S × M × L	8.47	0.132	0.46	Anecdotal
Bacterial abundance	S	14.98	< 0.001	> 150	Decisive
	M	15.26	0.009	31.67	Very strong
	L	0.03	0.859	0.53	Anecdotal
	S × M	22.20	< 0.001	94.44	Very strong
	S × L	0.03	0.864	1.77	Anecdotal
	M × L	8.02	0.155	2.01	Anecdotal
	S × M × L	9.22	0.100	0.95	Anecdotal
Fungal abundance	S	7.95	0.005	> 150	Decisive
	M	0.50	0.779	0.69	Anecdotal
	L	3.22	0.073	1.27	Anecdotal
	S × M	1.41	0.493	0.34	Anecdotal
	S × L	0.60	0.439	0.40	Anecdotal
	M × L	7.69	0.021	0.69	Anecdotal
	S × M × L	4.90	0.086	1.40	Anecdotal

### Effects of Light on Leaf Litter Decomposition

Our results indicate the occurrence of negative algal priming associated with alder leaf litter: in the presence of light, leaf mass loss was up to ~ 20% reduced in treatments that combine all microbial groups (bacteria, diatoms, and fungi) compared to the situation in absence of light, a finding that confirms an earlier study [11]. For beech, on the other hand, leaf mass loss was only marginally (~ 5%) altered in treatments that combine all microbial groups in presence compared to absence of light (Fig. 1), suggesting the absence of algal priming. These observations contradict with our hypotheses, namely that we observe positive priming under the low nutrient conditions tested here [8] and that recalcitrance of the leaf material should mediate priming intensity, leading to more pronounced priming on the more recalcitrant beech compared to the less recalcitrant alder leaf material.

**Fig. 2:**
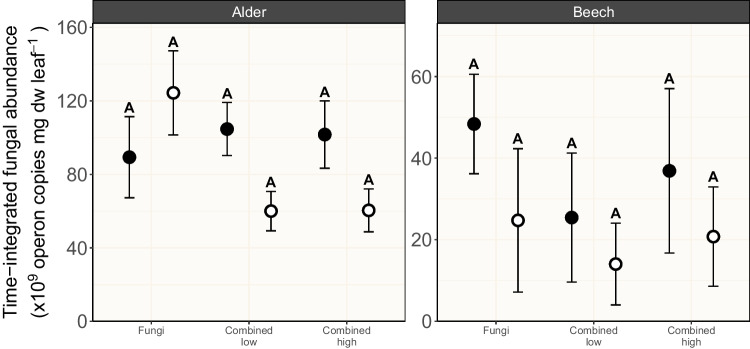
Time-integrated fungal abundance (using 10^9^ fungal operon copies mg dw leaf^−1^ as proxy; mean ± SE; *n* = 4–5) associated with black alder (left) and European beech (right) leaf material subjected to three microbial treatments in absence (filled circles) and in presence (open circles) of light. Letters above error bars denote results of pairwise comparisons among treatments, with different letters indicating statistically significant differences among treatments. Details on the microbial treatments are given in Table 1

Although we did not quantify algal exudates here, several earlier studies in aquatic ecosystems suggest that wide-ranging effects on leaf litter decomposition (enhancement or inhibition) can be explained by the presence of algal exudates that are considered as a source of labile C [see 11, 13]. Negative algal priming can mechanistically be explained by heterotrophs’ preferential use of diatom-derived labile C exudates over leaf-derived C, indirectly reducing heterotrophic decomposition of leaf litter [11, 13]. Nevertheless, fungi did not make use of the diatom-derived labile C exudates to increase their abundance; on the contrary, alder-associated fungi showed a ~ 40% decrease in abundance in the treatments that combine all microbial groups in presence relative to the absence of light (Fig. 2). Light itself is unlikely to cause the observed reduction in fungal abundance in the combined treatments, as fungal abundance increased by ~ 30% in the fungi-only treatment in presence compared to the absence of light (Fig. 2) [46]. The phenomenon of reduced fungal abundance when combining autotrophs and heterotrophs in presence of light was likewise found by Halvorsson et al. [11], who concluded that fungi do not invest algal-derived C into new hyphal growth. Instead, fungi channel energy rather to alternative (reproductive) pathways such as spore production that accounts for more than 80% of AH productivity in some species [47, 48]. Since we did not quantify AH sporulation during the present study, this assumption remains an open question that needs further scrutiny, especially given that we used laboratory AH cultures that may lose their ability to sporulate when maintained over the long term [49].

**Fig. 3:**
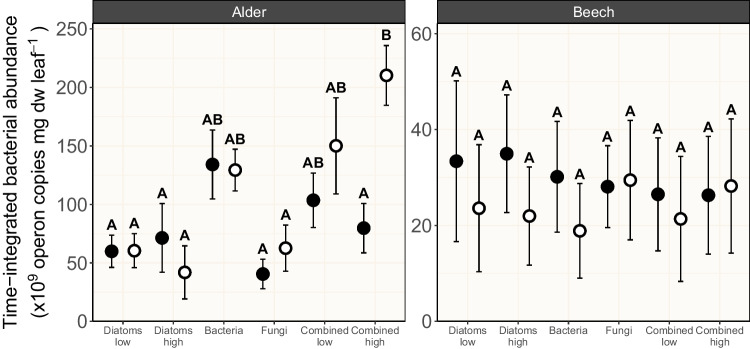
Time-integrated bacterial abundance (using 10^9^ bacterial operon copies mg dw leaf^−1^ as proxy; mean ± SE; *n* = 4–5) associated with black alder (left) and European beech (right) leaf material subjected to six microbial treatments in absence (filled circles) and in presence (open circles) of light. Letters above error bars denote results of pairwise comparisons among treatments, with different letters indicating statistically significant differences among treatments. Details on the microbial treatments are given in Table 1

Unlike fungi, bacteria apparently channeled the diatom-derived C into biomass accrual. When combining all microbial groups in presence of light, bacterial abundance increased by up to ~ 160% compared to the scenario without light, which even exceeded the abundance in the “bacteria” treatment by up to ~ 60% (Fig. 3). This steep increase in bacterial abundance in presence of light may be explained by the availability of labile C exudates created by diatoms [15, 50] and supports the assumption that bacteria grow rapidly when labile C is available [51]. Since we, however, did not measure diatom-derived labile C exudates in the water phase, this potential mechanism is speculative and requires experimental support in further studies. Nevertheless, the higher bacterial abundance seemingly did not translate into a higher bacterial activity in terms of leaf litter decomposition (Fig. 1), which could be triggered by the more easily available energy in the form of diatom exudates compared to the leaf-bound C.

### Effects of Leaf Species Identity on Leaf Litter Decomposition

**Fig. 4:**
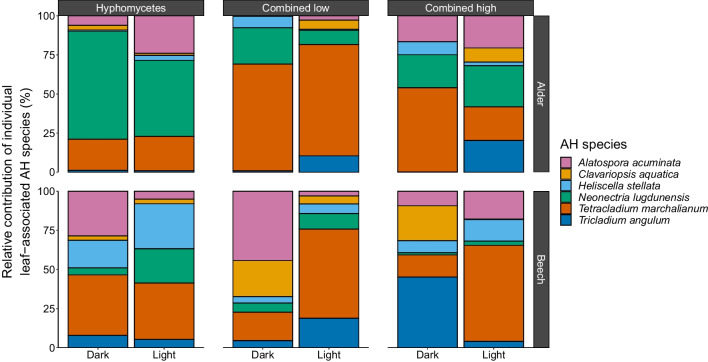
Mean relative contribution (%; *n* = 4–5) of individual aquatic hyphomycete (AH) species to the overall AH assemblages associated with black alder and European beech leaf material separated by microbial treatments containing AH (Table 1) in absence and in presence of light. Colors refer to the individual AH species (see legend). Absolute DNA amounts of the individual AH species can be found in Table S4

The decisive influence of the factor “leaf species identity” on leaf mass loss is likely mediated by the leaf materials’ stoichiometry and recalcitrance. As shown in previous studies, alder leaves contain ~ threefold more nutrients and ~ twofold less lignin than beech leaves [17]. Since microbial decomposers grow better on leaf material with a high content of labile carbon and nutrients [52], alder leaves were a better substrate for microbial colonization and activity. This assumption is underpinned by the time-integrated leaf-associated abundance of bacteria and fungi, which are both decisively affected by the leaf species identity (*χ*^2^ = 7.95, *p* = 0.005, BF_10_ > 150; *χ*^2^ = 14.98, *p* < 0.001, BF_10_ > 150; Table 2), with a higher abundance of both microbial groups on alder leaves (Figs. 2 and 3). Nevertheless, the sole abundance of leaf-associated fungi delivers less information about their functional potential than their composition, given that aquatic fungi differ in their functional traits [53]. As observed earlier [54], the AH composition associated with alder and beech leaves differed (*F*_1,48_ = 22.85, *p* = 0.001; Table 3), leading to distinct patterns of assemblage compositions (Fig. 4). In general, the litter species seemed to mediate the abundance of individual AH species on leaf litter [e.g., 55] and by this, their assemblage composition. In this context, the advanced decomposition of alder leaves compared to beech leaves may have allowed for an advanced succession of the fungal assemblages and niche differentiation due to resource partitioning [56], which allowed AH with high decomposition efficacies to co-exist. Our study revealed that *N. lugdunensis* and *T. marchalianum* were the species that contributed most (~ 51%; Table S5) to the differences in the alder and beech leaf-associated AH abundance (Fig. 4). Within the AH assemblage tested in the present study, the formerly mentioned species are among those with the highest efficacy to decompose leaf material, both individually and in combination [53]. Consequently, the higher abundance of *N. lugdunensis* and *T. marchalianum* associated with alder leaves should at least partially explain the decisive influence of leaf species identity on leaf mass loss. Such mechanisms are likewise conceivable for bacterial communities, as they are functionally more diverse than fungi [57] and at least play a minor role in leaf litter decomposition [4]. Nevertheless, our limited knowledge on microbial traits hinders a more detailed interpretation of the structural and functional responses and calls for further research.

**Table 3:** Output for the permutational analysis of variance testing the effects of leaf species (S), microbial treatment (M), and light condition (L) on the relative abundance of the leaf-associated aquatic hyphomycete assemblages

Factor	Df^1^	SumSq^2^	*F*-value	*p*-value
S	1	0.436	22.85	0.001
M	2	0.075	1.96	0.148
L	1	0.040	2.09	0.001
S × M	2	0.102	2.68	0.033
S × L	1	0.153	8.03	0.005
M × L	2	0.065	2.08	0.030
S × M × L	2	0.079	2.04	0.362
Residual	36	0.687		
Total	48	1.651		

^1^Degrees of freedom

^2^Sum of squares

### Treatment Effects on and Interactions Among Microbial Groups

We could not detect leaf-associated diatoms in any of the treatments (Table 1) using HPLC (irrespective of the light condition; all measurements have been below the level of detection). In contrast, diatoms could be detected in other habitats, namely the medium after scraping off the microcosms’ glass walls, with mean (± standard error) concentrations of up to 0.34 (± 0.07) µg fucoxanthin L^−1^ in all treatments containing diatoms in the presence of light. Depending on the microbial treatment, these results may be explained by several mechanisms: first, heterotrophs being better competitors for inorganic nutrients than autotrophs [14]. In fact, the growth of autotrophs can be strongly dampened in the presence of heterotrophs in combination with low phosphorous (P) availability [58], as also simulated in the present study. Second, in addition to nutrient competition, autotrophic and heterotrophic microorganisms compete for space through the access to a substrate attachment zone. Such spatial competition can add a supplementary selective pressure that may cause allelopathic interactions among autotrophic and heterotrophic microorganisms [59, 60] potentially inhibiting algal growth in treatments with all microbial groups present. Third, diatoms require silica to build up their ornamented cell walls [61]. An adhesion of diatoms to the glass is supported by the diatoms capability to extract silica from glass [62]. Fourth, the water velocity created by the aeration system created strong turbulence in the microcosms that may have hindered diatom adhesion on the leaf substrate. Consequently, a set-up which allows diatoms to colonize leaf material before initiating aeration could have stimulated their permanent adhesion to leaf material. Although such set-ups would have benefitted the assessment of mechanisms, they would not fully reflect the environmental conditions of headwater streams (e.g., fast water flow) where autotrophic and heterotrophic microorganisms predominantly compete for resources such as nutrients and substrates [63].

The time-integrated bacterial abundance was very strongly affected by the microbial treatment (*χ*^2^ = 15.26, *p* = 0.009, BF_10_ = 31.67), but this effect was very strongly dependent on the leaf species (*χ*^2^ = 22.20, *p* < 0.001, BF_10_ = 94.44) as indicated by the interaction between these factors. While the beech leaf-associated bacterial abundance varied around one mean value irrespective of the microbial treatment and light condition, treatment and light effects became visible for bacteria associated with alder leaves (Fig. 3). The occurrence of bacteria in microbial treatments without active bacterial dosing is likely explained by the fact that we did not autoclave the leaf material before its use in the experiments. Autoclaving is assumed to structurally alter the leaf material, resulting in leaching of water-soluble compounds and nutrients [64], which may have influenced the results of the present study. Therefore, we likely introduced some leaf-associated bacteria into the “diatom” and “fungi” treatments that thrived over the course of the experiment.

In absence of light, the highest and lowest bacterial abundances associated with alder leaves were observed for the “bacteria” treatment and the “fungi” treatment, respectively (~ 230% difference), likely stemming from the active bacterial dosing in the “bacteria” treatment. When combining all microbial groups, a suppression of bacteria by fungi [65, 66] became visible in absence of light, indicated by an up to 70% reduction in bacterial abundance in the “combined low” and “combined high” treatment relative to the “bacteria” treatment (Fig. 3). When growing on decomposing leaf litter, aquatic fungi and bacteria are in close spatial proximity, although fungal hyphae are able to penetrate the substrate, while bacterial growth is limited to the leaf surface (with the exception of tunneling bacteria) [67]. Therefore, leaf litter-associated aquatic fungi and bacteria interact with each other via various antagonistic mechanisms such as resource competition [65] and the release of secondary metabolites that may exert antibiotic characteristics [66]. The observed antagonism between heterotrophs seemed to be repealed by the diatom exudates in presence of light that allowed bacteria to thrive substantially.

**Fig. 5:**
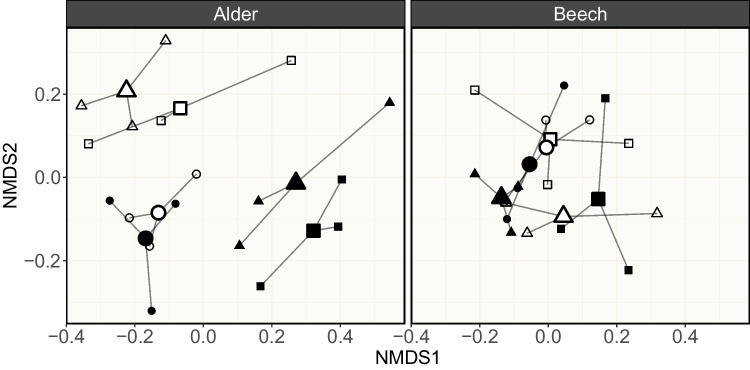
Nonmetric multidimensional scaling (NMDS) ordination plots for AH assemblages associated with black alder and European beech leaf material (*n* = 3). Symbols indicate the microbial treatment and light condition: filled circles (“fungi” in absence of light), open circles (“fungi” in presence of light), filled squares (“combined low” in absence of light), open squares (“combined low” in presence of light), filled triangles (“combined high” in absence of light), and open triangles (“combined high” in presence of light; for details on the microbial treatments, see Table 1). In addition, the group centroids (large symbols) of the individual treatments are shown, which connect the respective replicates via spider webs. The stress values of the NMDS ordinations were below 0.2 (black alder = 0.12, European beech = 0.08), indicating a reasonable fit of the data [43]

For the AH assemblage composition, we observed an influence of leaf species identity (*F*_2,47_ = 22.85, *p* = 0.001), which showed an interaction with the microbial treatment (*F*_5,47_ = 2.68, *p* = 0.033) and the applied light condition (*F*_2,47_ = 8.03, *p* = 0.005). While we could not abstract a clear assemblage pattern for beech leaf-associated AH under the influence of the applied microbial treatments or the light conditions, the opposite became visible for alder leaf-associated AH (Fig. 5): the NMDS ordination revealed that light did not affect the AH assemblages in the treatments with no other microbial groups present. Contrarily, light showed a clear influence on the AH assemblages in the treatments with all microbial groups present. Identifying the exact mechanism for the difference in individual AH thriving in the microbial treatments and under the differing light conditions is challenging, but previous research points to several possibilities. While light seemingly did not influence fungal growth in the treatment with only fungi present, given the similarity in the relative contribution of AH species (Fig. 4) and the close proximity of group centroids in the ordination (Fig. 5), the simultaneous presence of diatoms and light in the “combined” treatments apparently created different ecological niches for fungi. First, AH species may have differed in their use of the diatom-derived labile C and could invest a lower or higher share of available energy into reproduction and growth, as suggested under the dynamic energy budget theory [68]. Second, light could have acted as a filter [69] that selects individual species based on their traits, favoring their survival and growth. Third, allelopathic inhibition among microbial groups [70, 71] may have reduced the abundance of weak competitors and shaped the observed AH assemblages. Nevertheless, given that knowledge on microbial traits such as carbon usage, light preference and competitive strength is limited, we require a consolidated effort to develop respective data bases informing the interpretation of studies such as ours. Such data could be generated by designing experiments that make use of methodological advances to track the contribution of individual fungal species to the fungal communities’ composition and activity [53]. These experiments would simultaneously advance our understanding of the biodiversity-ecosystem functioning relationship by revealing how the interaction among fungal species affects the overall performance of fungal communities through complementarity and selection effects and by this the net biodiversity effect.

## Conclusion

Our findings of negative algal priming point to unexpected effects on litter C processing in stream food webs. The immediate effect of negative algal priming may be a shift of heterotrophic decomposers in their utilization of terrestrially-derived leaf litter from using it as a C source to a surface substratum for sole growth. Such a conversion in leaf litter use inevitably slows down the litter processing chain, leading to increased C storage in the form of coarse particulate organic matter (CPOM) and a potential CPOM export downstream. On the contrary, reduced litter processing in headwater streams likely results in a reduced downstream transport of fine particulate organic matter (FPOM), which has particular consequences for the longitudinal connectivity in stream ecosystems [63]. Furthermore, since we observed that algal-derived C was not utilized to build up fungal biomass, such labile C may only poorly be transferred to higher trophic levels.

Nevertheless, since our study was conducted at the microcosm scale, the interactions and implications observed here need to be confirmed in situ, as microbial interactions and their effects may be patchier and persist only over short time (days to weeks) under field conditions [11]. In addition, study designs with more complex microbial assemblages are needed for a deeper understanding of microbial interactions and their mechanisms—both at the microcosm and field scale—and as such of the direction and implications of autotrophic priming in aquatic systems.

### Supplementary Information

Below is the link to the electronic supplementary material.

Supplementary file1 (DOCX 141 KB)

## Data Availability

The data that support the findings of this study are openly available in GitHub at https://github.com/aflandau/https-doi.org-10.1007-s00248-023-02268-w. Data, associated metadata, and calculation tools are also available from the corresponding author upon reasonable request (alexander.feckler@rptu.de).
