# Supplementary material for: Diatoms Reduce Decomposition of and Fungal Abundance on Less Recalcitrant Leaf Litter via Negative Priming
Source: Microb Ecol. 2023 Jul 28;86(4):2674–86. doi: 10.1007/s00248-023-02268-w (PMC10640500; doi:10.1007/s00248-023-02268-w)
Supplement: Supplementary file 1 — Supplementary file1 (DOCX 141 KB) [file 248_2023_2268_MOESM1_ESM.docx]

**Supporting Information for the paper:**

**Diatoms reduce decomposition of and fungal abundance on less recalcitrant leaf litter via negative priming**

Alexander Feckler^1,2,3,^*, Patrick Baudy-Groh^1^, Lisa Friedrichs^1^, Sara Gonçalves^1^, Simon Lüderwald^1^, Ute Risse-Buhl^4^, Mirco Bundschuh^1,2^

^1^iES Landau, Institute for Environmental Sciences, RPTU Kaiserslautern-Landau, Fortstraße 7, D-76829 Landau, Germany

^2^Department of Aquatic Sciences and Assessment, Swedish University of Agricultural Sciences, Box 7050, SWE-75007 Uppsala, Sweden

^3^Eußerthal Ecosystem Research Station, RPTU Kaiserslautern-Landau, Birkenthalstraße 13, 76857 Eußerthal, Germany

^4^Department of River Ecology, Helmholtz Centre for Environmental Research – UFZ, Brückstraße 3a, 39114 Magdeburg, Germany

**COMPOSITION OF THE TEST MEDIUM**

Table S1 Composition of the test medium adapted after [1] and [2]. The pH was adjusted to 7 using 2 M NaOH immediately after medium preparation

| Salt | Concentration (mg L^-1^) |
| --- | --- |
| ***Major nutrients*** |  |
| CaCl_2_ × 2H_2_O | 100.00 |
| MgSO_4_ × 7H_2_O | 10.0 |
| Morpholino propane sulfonic acid | 500.0 |
| KNO_3_ | 1.44 |
| K_2_HPO_4_ | 0.11 |
| Na_2_SiO_3_ × 9H_2_O | 0.57 |
| ***Trace elements*** |  |
| Na_2_ × EDTA | 0.436 |
| FeCl_3_ × 6H_2_O | 0.315 |
| CuSO_4_ × 5H_2_O | 0.001 |
| ZnSO_4_ × 7H_2_O | 0.0022 |
| CoCl_2_ × 6H_2_O | 0.001 |
| MnCl_2_ × 4H_2_O | 0.018 |
| Na_2_MoO_4_ × 2H_2_O | 0.0006 |
| H_3_BO_3_ | 0.1 |

**INFORMATION ON TaqMan^®^ PROBE-BASED qPCR ASSAYS USED DURING THE STUDY**

Table S2 Information on qPCR assay designations, targeted species, including the used model strain and template sequences as well as technical properties including melting temperature, guanine-cytosine content, binding region, amplicon length, limits of detection as well as quantification (expressed as cycle of quantification (C_q_) and DNA concentration per PCR), and quantitative ranges covered by DNA standards [3]

| qPCR assay designation | Target species | Target strain (DSM number) | Template ITS sequence  (GenBank accession number) | Primer/ probe | Sequence (5'-3') | Primer/ probe length (bp) | Melting temperature (°C) | guanine-cytosine content (%) | Binding region | Amplicon length (bp) | Limit of detection | |  | Limit of quantification | | Quantitative standards covered by the assay (ng/µL) |
| --- | --- | --- | --- | --- | --- | --- | --- | --- | --- | --- | --- | --- | --- | --- | --- | --- |
|  |  |  |  |  |  |  |  |  |  |  | C_q_ value | fg/PCR |  | C_q_ value | fg/PCR |  |
| ALAC-tqmn | *Alatospora acuminata* | DSM 104360 | MH930815 | Forward | CGTAGTAATTTTCTCGCTTTGGAGA | 21 | 59.0 | 52 | *ITS2* | 82 | 39.04 | 3.57 |  | 34.50 | 65.22 | 10-10^-5^ |
|  |  |  |  | Reverse | CCTGATCCGAGGTCAACCTTT | 21 | 59.0 | 52 | *ITS2*/*LSU* | |  |  |  |  |  |  |
|  |  |  |  | Probe | 6 FAM-TTTGCCAACAACCC-MGBNFQ | 14 | 68.0 | 50 | *ITS2* |  |  |  |  |  |  |  |
| CLAQ-tqmn | *Clavariopsis aquatica* | DSM 104362 | MH930817 | Forward | AATTCATTGGCAGCCGGTAA | 20 | 59.0 | 45 | *ITS2* | 82 | 40.08 | 0.65 |  | 35.79 | 11.56 | 10-10^-5^ |
|  |  |  |  | Reverse | AAGAGGCTTGATGGAAGGAGGTA | 23 | 59.2 | 48 | *ITS2* |  |  |  |  |  |  |  |
|  |  |  |  | Probe | 6 FAM-TGTGTCGCGATCCAGT-MBGNFQ | 16 | 70.0 | 56 | *ITS2* |  |  |  |  |  |  |  |
| HEST-tqmn | *Heliscella stellata* | DSM 104386 | MH930820 | Forward | GCGTCGTAGTTTGACCAAGACA | 22 | 58.9 | 50 | *ITS2* | 79 | 41.41 | 7.71 |  | 37.05 | 142.13 | 10-10^-4^ |
|  |  |  |  | Reverse | AAACAGATGATTCAAGGCTAATCGT | 25 | 58.3 | 36 | *ITS2* |  |  |  |  |  |  |  |
|  |  |  |  | Probe | 6 FAM-CAATGTGAAACTAAGTCAGATAA-MGBNFQ | 23 | 70.0 | 30 | *ITS2* |  |  |  |  |  |  |  |
| NELU-tqmn | *Neonectria lugdunensis* | DSM 104361 | MH930822 | Forward | TGTAGCTTCCTCTGCGTAGTAGCA | 24 | 59.0 | 50 | *ITS2* | 90 | 38.02 | 3.52 |  | 33.73 | 62.56 | 10-10^-5^ |
|  |  |  |  | Reverse | CCGAGGTCAACCTTTCAGAAGT | 22 | 58.0 | 50 | *ITS2*/*LSU* | |  |  |  |  |  |  |
|  |  |  |  | Probe | 6 FAM-TCGCACTGGAAAGC-MGBNFQ | 14 | 69.0 | 57 | *ITS2* |  |  |  |  |  |  |  |
| TEMA-tqmn | *Tetracladium marchalianum* | DSM 104373 | MH930823 | Forward | GCTGTCAGGCTCTAAGCGTAGTAA | 24 | 58.0 | 50 | *ITS2* | 64 | 39.11 | 2.03 |  | 34.75 | 36.16 | 10-10^-5^ |
|  |  |  |  | Reverse | GTTCTGGCGAGTGTCCATCA | 20 | 58.0 | 55 | *ITS2* |  |  |  |  |  |  |  |
|  |  |  |  | Probe | 6 FAM-CTCTCTCGCTACAGACAC-MGBNFQ | 18 | 69.0 | 56 | *ITS2* |  |  |  |  |  |  |  |
| TRAN-tqmn | *Tricladium angulatum* | DSM 104374 | MH930824 | Forward | CCTGTTCGAGCGTCATCAAA | 20 | 58.5 | 50 | *5.8S*/*ITS2* | 129 | 42.16 | 0.72 |  | 37.68 | 13.36 | 10-10^-5^ |
|  |  |  |  | Reverse | CCGACGTCTATAGCGAGAAGAATT | 24 | 59.0 | 46 | *ITS2* |  |  |  |  |  |  |  |
|  |  |  |  | Probe | 6 FAM-CCTGGCAGCCCTTA-MGBNFQ | 14 | 68.0 | 64 | *ITS2* |  |  |  |  |  |  |  |

**INFORMATION ON ‘MODEL AMPLICONS’ OF *TETRACLADIUM MARCHALIANUM* AND *ESCHERICHIA COLI***

Table S3 Sequences of *Escherichia coli* (Migula 1895) Castellani and Chalmers 1919 and *Tetracladium marchalianum* de Wild. ‘model amplicons’, their length (base pairs), and source of the sequences that were used for estimations of fungal and bacterial operon copies, respectively. Primer binding sites are highlighted in bold and are underlined

| Species | Sequence | Length (bp) | Source |
| --- | --- | --- | --- |
| *E. coli* | AAATTGA**AGAGTTTGATCATGGCTCAG**ATTGAACGCTGGCGGCAGGCCTAACACATGCAAGTCGAACGGTAACAGGAAGAAGCTTGCTTCTTTGCTGACGAGTGGCGGACGGGTGAGTAATGTCTGGGAAACTGCCTGATGGGCCTCTTGCCATCGGATGTGCCCAGATGGGATTAGCTAGTAGGTGGGGTAACGGCTCACCTAGGCGACGATCCCTAGCTGGTCTGAGAGGATGACCAGCCACACTGGAACTGAGACACGGTCCAGACTCCTACGGGACGCAGCAGTGGGGAATATTGCACAATGGGCGCAAGCCTGATGCAGCCATGCCGCGTGTATGAAGAAGGCCTTCGGGTTGTAAAGTACTTTCAGCGGGGAGGAAGGGAGTAAAGTTAATACCTTTGCTCATTGACGTTACCCGCAGAAGAAGCACCGGCTAACTCC**GTGCCAGCAGCCGCGGTAA**CTCCGTG | 470 | [4] |
| *T. marchalianum* | CTTGGCTCTG**GCATCGATGAAGAACGCAGC**GAAATGCGATAAGTAATGTGAATTGCAGAATTCAGTGAATCATCGAATCTTTGAACGCACATTGCGCCCCTTGGTATTCCGAGGGGCATGCCTATTCGAGCGTCATTATCACCCCTCAAGCTCAGCTTGGTGTTGAGGCCTGCTGTCAAGGCAGCCTCTAAAAGCAGTGGCAGTGCTGTCAGGCTCTAAGCGTAGTAATCTCTCTCGCTACAGACACCTGATGGACACTCGCCAGAACCCCCCATCTTTTAATGATTGACCTCGGATTAGGTAGGGATACCCGCTGAACTTAA**GCATATCAATAAGCGGAGGA**AAAGAAACCA | 353 | Sequence of *T. marchalianum* strain DSM 104373  (GenBank accession no. MH930823) |

**EXEMPLARY MELTING CURVE FOR SYBR GREEN-BASED qPCR ANALYSES**

We carried out melting curve analyses for SYBR Green-based qPCR assays (estimations of bacterial and fungal operon copies) to test the assays’ specificity. This was achieved by initial denaturation of the qPCR products for 15 sec at 95 °C, followed by a steady temperature increase for 20 min from 60 °C to 95 °C. The defined peak in Figure S1 at around 87 °C in all analyzed samples (except the negative controls) suggests a clean amplification of a single product. As this pattern was consistently observed for all qPCR runs, we conclude that the used method was specific for the designated DNA motifs.


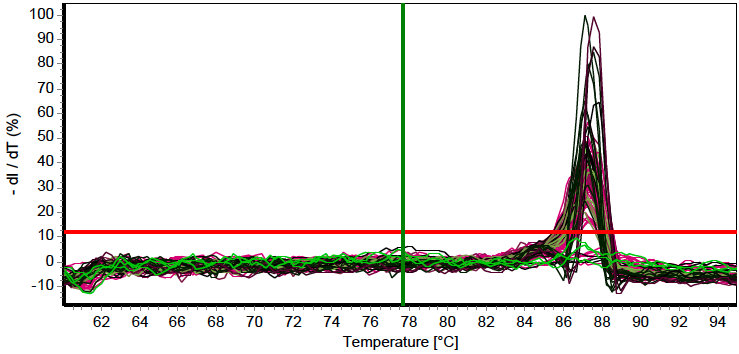


Fig. S1 Exemplary melting curve plot for a SYBR Green-based analysis of bacterial operon copy number showing the percentage reduction in fluorescence intensity with time over a temperature gradient from 60 °C to 95 °C

**ABSOLUTE DNA AMOUNTS OF AQUATIC HYPHOMYCETE SPECIES AT THE INDIVIDUAL SAMPLING POINTS**

Table S4 Absolute DNA amounts (fg mg dw leaf^-1^; ± standard error; *n* = 5) of individual aquatic hyphomycete species, separated by leaf species, microbial treatments, and sampling points. Details of the microbial treatments are given in Table 1

| Leaf species | Light | Mircobial treatment | Time (d) | *A. acuminata* | *C. aquatica* | *H. stellata* | *N. lugdunensis* | *T. marchalianum* | *T. angulatum* |
| --- | --- | --- | --- | --- | --- | --- | --- | --- | --- |
| Black alder | Absent | Fungi | 10 | 0.12 ± 0.07 | 0.77 ± 0.45 | 0.01 ± 0.01 | 0.98 ± 0.69 | 0.50 ± 0.23 | 0.01 ± 0.01 |
|  |  | Combined low | 10 | 0.00 ± 0.00 | 0.12 ± 0.08 | 0.08 ± 0.03 | 0.06 ± 0.04 | 7.01 ± 5.51 | 0.02 ± 0.01 |
|  |  | Combined high | 10 | 0.00 ± 0.00 | 0.15 ± 0.14 | 0.10 ± 0.06 | 0.08 ± 0.06 | 10.61 ± 8.8 | 0.02 ± 0.01 |
|  | Present | Fungi | 10 | 2.71 ± 2.70 | 0.11 ± 0.04 | 0.01 ± 0.01 | 4.22 ± 3.77 | 0.69 ± 0.52 | 0.01 ± 0.01 |
|  |  | Combined low | 10 | 0.50 ± 0.40 | 0.03 ± 0.01 | 0.03 ± 0.03 | 0.40 ± 0.35 | 0.05 ± 0.05 | 0.02 ± 0.02 |
|  |  | Combined high | 10 | 3.80 ± 2.60 | 2.90 ± 2.09 | 0.01 ± 0.01 | 0.44 ± 0.21 | 13.25 ± 13.22 | 0.00 ± 0.00 |
|  | Absent | Fungi | 20 | 0.45 ± 0.42 | 0.12 ± 0.04 | 0.05 ± 0.05 | 1.11 ± 0.42 | 0.95 ± 0.60 | 0.02 ± 0.01 |
|  |  | Combined low | 20 | 1.84 ± 1.84 | 0.00 ± 0.00 | 0.20 ± 0.14 | 5.25 ± 4.92 | 4.96 ± 3.41 | 0.06 ± 0.05 |
|  |  | Combined high | 20 | 0.25 ± 0.25 | 0.04 ± 0.03 | 0.60 ± 0.34 | 0.45 ± 0.26 | 3.42 ± 2.2 | 0.01 ± 0.01 |
|  | Present | Fungi | 20 | 1.66 ± 1.45 | 0.09 ± 0.06 | 0.11 ± 0.07 | 1.14 ± 0.73 | 24.80 ± 22.41 | 0.06 ± 0.04 |
|  |  | Combined low | 20 | 4.34 ± 3.53 | 0.03 ± 0.01 | 0.05 ± 0.03 | 9.21 ± 9.18 | 0.05 ± 0.04 | 0.01 ± 0.01 |
|  |  | Combined high | 20 | 0.41 ± 0.36 | 0.13 ± 0.08 | 0.01 ± 0.01 | 0.03 ± 0.02 | 79.08 ± 78.84 | 0.02 ± 0.01 |
|  | Absent | Fungi | 30 | 1.48 ± 1.45 | 0.04 ± 0.02 | 0.01 ± 0.01 | 4.70 ± 3.47 | 0.48 ± 0.26 | 0.15 ± 0.13 |
|  |  | Combined low | 30 | 0.01 ± 0.01 | 0.03 ± 0.01 | 0.33 ± 0.14 | 1.53 ± 1.42 | 202.15 ± 142.57 | 0.02 ± 0.02 |
|  |  | Combined high | 30 | 0.86 ± 0.52 | 0.004 ± 0.004 | 0.27 ± 0.25 | 0.05 ± 0.05 | 14.14 ± 9.04 | 0.01 ± 0.01 |
|  | Present | Fungi | 30 | 0.49 ± 0.24 | 0.19 ± 0.17 | 0.19 ± 0.15 | 6.99 ± 3.11 | 3.92 ± 2.39 | 0.04 ± 0.02 |
|  |  | Combined low | 30 | 0.01 ± 0.01 | 6.25 ± 6.23 | 0.51 ± 0.50 | 6.74 ± 6.70 | 179.55 ± 114.26 | 8.75 ± 7.37 |
|  |  | Combined high | 30 | 1.43 ± 1.09 | 13.50 ± 13.50 | 0.05 ± 0.03 | 1.65 ± 1.46 | 29.09 ± 28.81 | 0.1 ± 0.01 |

Table S4 continued

| Leaf species | Light | Mircobial treatment | Time (d) | *A. acuminata* | *C. aquatica* | *H. stellata* | *N. lugdunensis* | *T. marchalianum* | *T. angulatum* |
| --- | --- | --- | --- | --- | --- | --- | --- | --- | --- |
| European beech | Absent | Fungi | 10 | 27.66 ± 19.49 | 1.37 ± 1.35 | 4.76 ± 2.20 | 1.08 ± 0.36 | 40.77 ± 16.17 | 35.18 ± 14.55 |
|  |  | Combined low | 10 | 2.70 ± 2.37 | 4.57 ± 3.32 | 0.92 ± 0.51 | 3.68 ± 2.11 | 46.85 ± 20.94 | 24.60 ± 22.17 |
|  |  | Combined high | 10 | 1.98 ± 1.04 | 0.40 ± 0.23 | 0.96 ± 0.33 | 2.99 ± 1.58 | 24.76 ± 12.14 | 9.48 ± 4.86 |
|  | Present | Fungi | 10 | 7.73 ± 4.67 | 3.46 ± 3.35 | 0.44 ± 0.29 | 2.19 ± 1.44 | 38.14 ± 23.97 | 1.13 ± 1.04 |
|  |  | Combined low | 10 | 0.99 ± 0.74 | 3.00 ± 2.91 | 0.87 ± 0.36 | 0.84 ± 0.46 | 2.88 ± 2.26 | 11.22 ± 9.01 |
|  |  | Combined high | 10 | 7.17 ± 4.20 | 6.23 ± 5.53 | 2.20 ± 1.02 | 1.74 ± 0.37 | 31.16 ± 5.55 | 2.11 ± 1.67 |
|  | Absent | Fungi | 20 | 3.31 ± 1.75 | 1.07 ± 0.81 | 2.17 ± 1.43 | 0.46 ± 0.19 | 11.08 ± 5.34 | 3.42 ± 1.82 |
|  |  | Combined low | 20 | 28.71 ± 13.40 | 1.69 ± 1.65 | 3.06 ± 2.68 | 1.43 ± 0.83 | 57.83 ± 29.32 | 13.41 ± 7.83 |
|  |  | Combined high | 20 | 22.11 ± 19.73 | 0.61 ± 0.32 | 3.55 ± 2.79 | 1.33 ± 0.30 | 89.44 ± 39.08 | 11.27 ± 10.22 |
|  | Present | Fungi | 20 | 0.05 ± 0.04 | 0.09 ± 0.04 | 0.22 ± 0.14 | 0.56 ± 0.38 | 33.71 ± 31.28 | 0.42 ± 0.28 |
|  |  | Combined low | 20 | 9.51 ± 6.10 | 2.64 ± 1.32 | 1.02 ± 0.69 | 0.34 ± 0.16 | 45.44 ± 26.30 | 0.99 ± 0.92 |
|  |  | Combined high | 20 | 28.80 ± 13.71 | 29.60 ± 18.87 | 6.12 ± 2.78 | 3.74 ± 1.92 | 87.84 ± 8.98 | 59.93 ± 34.99 |
|  | Absent | Fungi | 30 | 7.67 ± 7.59 | 0.80 ± 0.58 | 2.67 ± 2.17 | 2.78 ± 1.24 | 21.76 ± 14.27 | 9.95 ± 9.46 |
|  |  | Combined low | 30 | 14.92 ± 5.29 | 2.66 ± 1.08 | 0.99 ± 0.37 | 1.06 ± 0.53 | 109.95 ± 64.77 | 0.16 ± 0.08 |
|  |  | Combined high | 30 | 28.29 ± 18.24 | 0.67 ± 0.59 | 2.53 ± 2.34 | 1.42 ± 0.69 | 70.34 ± 36.84 | 15.08 ± 12.14 |
|  | Present | Fungi | 30 | 2.56 ± 1.90 | 6.70 ± 6.13 | 0.91 ± 0.57 | 1.56 ± 0.69 | 27.13 ± 20.60 | 13.26 ± 12.88 |
|  |  | Combined low | 30 | 4.12 ± 3.95 | 0.18 ± 0.12 | 0.90 ± 0.59 | 0.38 ± 0.14 | 14.76 ± 9.71 | 2.98 ± 1.56 |
|  |  | Combined high | 30 | 5.55 ± 4.11 | 6.12 ± 4.40 | 0.54 ± 0.21 | 1.31 ± 0.19 | 54.12 ± 18.43 | 0.40 ± 0.16 |

**RESULTS OF THE SIMILARITY PERCENTAGE (SIMPER) ANALYSIS**

Table S5 Results of the SIMPER analysis run to identify the aquatic hyphomycete (AH) species that contributed most to the observed dissimilarities in AH assemblages between leaf species (black alder and European beech). The percentage contribution (%) of individual AH species to the overall assemblage (separated by leaf species) as well as their percentage contribution (%) to the observed dissimilarity are reported

| AH species | Mean contribution to alder-associated assemblage (%) | Mean contribution to beech-associated assemblage (%) | Contribution to dissimilarity (%) |
| --- | --- | --- | --- |
| *N. lugdunensis* | 27.5 | 4.4 | 27.3 |
| *T. marchalianum* | 41.3 | 47.4 | 23.3 |
| *T. angulatum* | 2.6 | 15.5 | 16.4 |
| *A. acuminata* | 15.3 | 15.4 | 14.0 |
| *C. aquatica* | 8.6 | 10.4 | 11.6 |
| *H. stellata* | 4.7 | 6.9 | 7.4 |

**References**

1. Dang CK, Chauvet E, Gessner MO (2005) Magnitude and variability of process rates in fungal diversity-litter decomposition relationships. Ecology Letters 8:1129–1137. https://doi.org/10.1111/j.1461-0248.2005.00815.x

2. Guillard RRL, Lorenzen CJ (1972) Yellow-green algae with chlorophyllide C1,2. Journal of Phycology 8:10–14. https://doi.org/10.1111/j.1529-8817.1972.tb03995.x

3. Baudy P, Zubrod JP, Röder N, et al (2019) A glance into the black box: Novel species-specific quantitative real-time PCR assays to disentangle aquatic hyphomycete community composition. Fungal Ecology 42:100858. https://doi.org/10.1016/j.funeco.2019.08.002

4. Baker GC, Smith JJ, Cowan DA (2003) Review and re-analysis of domain-specific 16S primers. Journal of Microbiological Methods 55:541–555. https://doi.org/10.1016/j.mimet.2003.08.009
